# Multi-Spectral Fusion and Denoising of Color and Near-Infrared Images Using Multi-Scale Wavelet Analysis †

**DOI:** 10.3390/s21113610

**Published:** 2021-05-22

**Authors:** Haonan Su, Cheolkon Jung, Long Yu

**Affiliations:** School of Electronic and Engineering, Xidian University, No. 2 South Taibai Road, Xi’an, Shaanxi 710071, China; zhengzk@xidian.edu.cn (C.J.); lyu@stu.xidian.edu.cn (L.Y.)

**Keywords:** image fusion, wavelet decomposition, color enhancement, near-infrared, denoising

## Abstract

We formulate multi-spectral fusion and denoising for the luminance channel as a maximum a posteriori estimation problem in the wavelet domain. To deal with the discrepancy between RGB and near infrared (NIR) data in fusion, we build a discrepancy model and introduce the wavelet scale map. The scale map adjusts the wavelet coefficients of NIR data to have the same distribution as the RGB data. We use the priors of the wavelet scale map and its gradient as the contrast preservation term and gradient denoising term, respectively. Specifically, we utilize the local contrast and visibility measurements in the contrast preservation term to transfer the selected NIR data to the fusion result. We also use the gradient of NIR wavelet coefficients as the weight for the gradient denoising term in the wavelet scale map. Based on the wavelet scale map, we perform fusion of the RGB and NIR wavelet coefficients in the base and detail layers. To remove noise, we model the prior of the fused wavelet coefficients using NIR-guided Laplacian distributions. In the chrominance channels, we remove noise guided by the fused luminance channel. Based on the luminance variation after fusion, we further enhance the color of the fused image. Our experimental results demonstrated that the proposed method successfully performed the fusion of RGB and NIR images with noise reduction, detail preservation, and color enhancement.

## 1. Introduction

In low light conditions, the captured RGB images are degraded with serious noise. Although many denoising methods [1,2,3] have been proposed and have obtained good performance in noise reduction, the performance on low light images requires improvement due to complicated noise modeling after a series of operations in the camera processing pipeline. Recent advances in multi-spectral imaging provide techniques to capture near infrared (NIR) and RGB images simultaneously [4,5]. As NIR images provide fine details and clear structure in the challenging condition, this technique is applied to a lot of multi-spectral image restorations, such as image dehazing [6], contrast enhancement [7], and image denoising [8].

In low light conditions, NIR cameras and dark flash are used to capture NIR images [9]. The dark flash projects light containing NIR and visible bands, and the visible band is blocked. NIR cameras are sensitive to the NIR spectral band with the range of 700 to 1100 nm [10]. In low light conditions, NIR images have the advantages of texture rendering without noise corruption compared to noisy RGB images because strong near-IR flash improves the NIR light reflection of the captured scenes, and thus NIR images capture more visible structures without noise corruption. On the other hand, RGB images contain better color information over NIR images. Therefore, NIR and RGB images were both employed to generate fused images with good structure, little noise, and vivid colors.

### 1.1. Related Work

#### 1.1.1. Gradient Processing

The basic idea of gradient-processing-based multi-spectral fusion is NIR images containing a clear structure without noise corruption compared to RGB images in low light conditions. Thus, researchers [8,11,12,13,14] propose multi-spectral image restoration guided by NIR gradients. However, RGB and NIR images have large discrepancies in gradients, which leads to blurred artifacts in fused images. Therefore, the correlation between the gradients of two images is applied to overcome the discrepancy problem. Zhuo et al. [11] developed dual weighted least square (WLS) smoothing, which employs two gradients of RGB and NIR images to remove the noise and preserve the main image structure in the luminance channel of RGB images.

Then, the structure from NIR images is transferred to denoised RGB images. However, the details of the results are also blurred with noise reduction. Li et al. [12] proposed blind image deblurring with the guidance of NIR image gradients. With a constraint on the difference between RGB and NIR image gradients, they generated clean results by conducting blur kernel estimation and image restoration alternatively. Shen et al. [8,13] introduced the gradient scale map (i.e., gradient ratio map) to deal with the discrepancy problem on two image gradients (e.g., gradient magnitude variation and gradient direction divergence between two images).

Using the scale map, they proposed two image restoration methods that denoise RGB images based on the common edges between two images. However, this leads to blurred artifacts when the edge magnitudes between two images were greatly different. Sugimura et al. [14] developed the simultaneous denoising and deblurring of low light images using several NIR images with a short exposure time.

They developed an energy function that used the gradient correlation between RGB and NIR images, between three RGB bands, and between temporal sequences to remove noise and improve the quality of the low light images. Yamashita et al. [4,5] developed a novel sensor that combined RGB and N sensors together, and they used this sensor to capture NIR and RGB raw data under low light conditions. Then, they employed traditional image demosaicing, motion deblurring, and denoising to extract RGB and NIR images, and enhanced the low light images with serious noise and blur.

#### 1.1.2. Image Fusion

In the first group of methods, we assumed that the NIR images contained more useful information without noise corruption compared with RGB images. This always occurs in texture regions that are highly corrupted in low light conditions. However, NIR images also contain distorted regions with low contrast and reduced details due to a different spectral sensitivity of NIR and RGB sensors [8]. The multi-spectral fusion guided by NIR images distorts the original structure of RGB images in fusion results. Thus, the idea of the second group of methods borrows from image fusion, which combines the most useful information from both the RGB and NIR images.

Image denoising is applied to fusion results or before image fusion. Son et al. [15] proposed the multi-spectral fusion of RGB and NIR images based on layer decomposition. They divided RGB and NIR images into base and detail layers. In the NIR base layer, they generated new NIR data, which had the same appearance as the RGB data and preserved the local contrast of NIR data with contrast preservation regularization. Then, they generated three detail layers (from the noisy RGB image, NIR image, and its new NIR data) and fused them. Then, the residual-based sparsity priors were applied to denoise the fused detail layer. However, blur artifacts and color noise still remained in the fusion results.

Son et al. [7] proposed a NIR coloring method for RGB image restoration. The proposed method consists of three steps: a contrast-preserving conversion that is the same as the new NIR data generation in  [15], detail fusion between RGB and NIR images, and color fusion from RGB colors. Shibata et al. [16] proposed multi-spectral image fusion using local contrast and inconsistent measurements. They estimated fusion weights based on the high visibility from two images and the prevention of artifacts from inconsistency. Li et al. proposed a novel encoder-decoder architecture consisting of convolutional layers extracting RGB and NIR image features, a fusion layer with two image features, and a dense block [17]. They introduced a dense block to reuse features from the middle layer for the reconstruction layer.

Zhang et al. proposed a novel fusion method based on the Pulse Coupled Neural Network (PCNN) in a Non-Subsampled Shearlet Transform (NSST) [18]. The adaptive parameters of PCNN were set by the inherent characteristics (i.e., the gradient energy and the magnitude of NSST coefficients) of images. Zheng et al. proposed a novel image fusion for haze removal using a set of artificial multi-exposure images that were generated by gamma correction [19]. They selected the best visual quality regions for fusion based on the entropy of the image texture.

Jung et al. proposed an unsupervised deep image fusion network consisting of three steps: feature extraction from RGB and NIR images, feature fusion between two image features, and fusion image reconstruction [20]. The unsupervised loss function based on a structure tensor was utilized to constrain the contrast of the output images similar to that of the input images.

### 1.2. Contributions

In this paper, we propose a multi-spectral fusion and denoising (MFD) framework of RGB and NIR images using multi-scale wavelet analysis. We assumed that RGB and NIR images have the same spatial resolution with well calibration, which means that the paired images contain matched structures without geometry displacements. The RGB and NIR bands are perfectly geometrically overlapped. There are no shadows or object movement in them. This is because the RGB or NIR images were taken by switching two filters (i.e., the IR cut filter and visible cut filter in front of the camera sensor) [10].

Thus, object movement leads to unmatched objects between paired images under different capturing times. To deal with the discrepancy and noise problem in the fusion, we formulate two observation models: (1) the noise model: the RGB image are corrupted with the additive Gaussian noise; (2) the discrepancy model: the discrepancy is measured as the correlation between RGB and NIR images, and we call this a wavelet scale map. Based on two observed models, we formulate the MFD framework as a maximum a posteriori (MAP) estimation that conducts the wavelet scale map estimation and image fusion alternatively.

To estimate the wavelet scale map, we utilize the priors of the wavelet scale map and its gradients as the contrast preservation term and gradient denoising term, respectively. To estimate the fused wavelet coefficients, we model the fusion coefficients as the Laplacian distribution with adaptive scaling parameters for noise removal. We apply the MFD framework to the luminance channel. In the chrominance channels, we utilize the fused luminance as a guidance to remove the chroma noise and provide color enhancement based on the luminance variation after fusion. Experimental results demonstrate that the proposed method generated fusion images with reduced noise, preserved details, and saturated colors.

**Figure 1:**
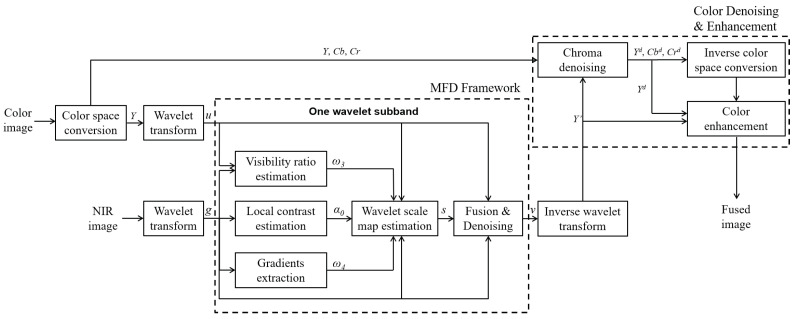
The entire framework of the proposed method (MFD: Multi-spectral fusion and denoising).

Figure 1 illustrates the entire diagram of the proposed method. In Figure 1, *Y*, Cb, and Cr are the luminance and chrominance channels after color space conversion; $Y^{\prime }$ is the fused luminance channel in the spatial domain after the inverse wavelet transform; *g* and *u* are the wavelet coefficients of NIR images and the luminance channel from RGB images in each subband; $\omega_{3}$ is the visibility ratio of NIR data to RGB data in (14); $\omega_{4}$ is composed of $\omega_{4x}$ and $\omega_{4y}$ in (15) and (16); $\alpha_{0}$ is the local contrast of NIR wavelet coefficients; *s* is the wavelet scale map; *v* is the wavelet coefficients in MFD; and $Y^{d}$, $Cb^{d}$, and $Cr^{d}$ are denoised luminance and chrominance channels.

Compared to our previous work [21], we have four extensions: (1) We introduce two observation models of noise and discrepancy from RGB data, NIR data, and the wavelet scale map in problem formulation. These models provide the mathematical basis for Bayesian derivation. (2) We apply the MFD to only the luminance channel. We perform guided filtering to two chroma channels and color enhancement based on the luminance variation after fusion. This can save the computational cost in the other two channels and generate results with vivid colors. (3) We provide a simplified MFD framework for the low frequency band to generate the results with a more visible structure of the NIR component in the base layer. (4) We formulate the prior of the fused wavelet coefficients as the adaptive Laplacian distribution using the guidance of NIR data. This provides good performance in detail preservation.

Compared with existing methods, the main contributions of the proposed method are as follows:

- We propose the MFD framework for RGB and NIR image fusion in the wavelet domain to achieve both texture transfer and noise removal.
- We provide the discrepancy model based on the wavelet scale map (correlation between RGB and NIR data) to deal with the discrepancy between RGB and NIR images.
- We combine three probability terms of contrast preservation, gradient denoising, and fusion denoising into the MFD framework to resolve the discrepancy while reducing the noise in the fusion.
- We enhance the color based on the luminance variation after fusion. The enhanced colors are more vivid with less color distortion.

The remainder of this paper is as follows. We describe the details of the proposed method in Section 2, while we provide the experimental results and their corresponding analysis in Section 3. We draw conclusions of this paper in Section 4.

## 2. Proposed Method

### 2.1. Problem Formulation

In this work, we aim to generate a high quality image by fusion and denoising from noisy RGB and NIR images. We first normalize the color bands by the maximum pixel value 255 (8-bit image). We decompose the Y channel of noisy RGB and NIR images using wavelet analysis. We denote the RGB wavelet coefficients in one subband as the vector u with *N* elements, the NIR wavelet coefficients in one subband as the diagonal matrix g with N×N elements, where the diagonal elements are NIR wavelet coefficients, and the fusion image as the vector v with *N* elements in the wavelet domain. The relationship between RGB, NIR and fusion images are formulated as follows:

(1) $$\begin{array}{ccc} u & = & v+N_{1} \end{array}$$

(2) $$\begin{array}{ccc} v & = & g\alpha +N_{2} \end{array}$$

where the vector $N_{1}$ represents the random Gaussian noise with *N* elements, the vector α with *N* elements is defined as wavelet scale map to model the correlation between RGB and NIR images, and the vector $N_{2}$ means the random error with *N* elements. (1) represents the noise model. That means the observed noisy RGB wavelet component u is generated from the desired RGB wavelet component v with noise $N_{1}$. (2) represents the discrepancy model. The relationship between RGB and NIR data are modeled as a linear function.

### 2.2. Multi-Spectral Fusion and Denoising Framework

**Figure 2:**
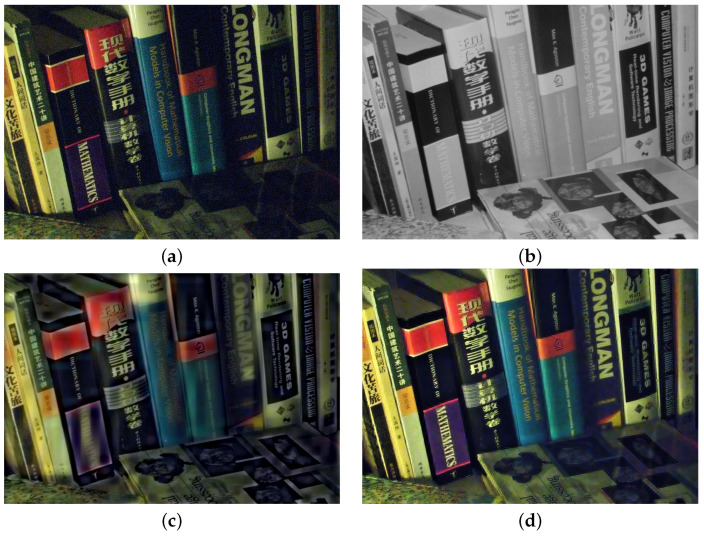
Fusion and denoising results in *Books* by Guided Wavelet Shrinkage (GWS) [1]. (**a**) RGB image. (**b**) NIR image. (**c**) GWS. (**d**) Proposed method.

We preform multi-spectral fusion and denoising (MFD) of NIR and RGB images based on multi-scale wavelet analysis and Bayesian theory [1]. Direct fusion of RGB and NIR images causes annoying artifacts, such as contrast attenuation due to the large discrepancy between two as shown in Figure 2c. Thus, we estimate a wavelet scale map to update the NIR wavelet coefficients and deal with the discrepancy between them. We formulate MFD as a maximum a posterior (MAP) estimation problem that finds clean RGB wavelet coefficients v and a wavelet scale map α given noisy RGB wavelet coefficients u and NIR wavelet coefficients g. For the MAP estimation, we maximize:

(3) $$\begin{array}{ccc} v,\alpha & = & \max_{v,\alpha }p\left(v,\alpha |u,g\right)\\ & = & \max_{v,\alpha }\left\{p\left(u,g|v,\alpha \right)\cdot p\left(v,\alpha \right)/p\left(u,g\right)\right\}\}\\ & \propto & \min_{v,\alpha }\left\{-\log p\left(u,g|v,\alpha \right)-\log p\left(v,\alpha \right)\right\} \end{array}$$

where p(v,α|u,g) is the posterior and p(u,g|v,α) is the likelihood; p(v,α) is the prior with joint distribution of v and α. p(u,g) has the same definition as p(v,α). p(u,g) is a constant and thus is omitted in the optimization.

First, we design the likelihood for the fusion of noisy RGB and NIR images based on the noise model and the discrepancy model in Section II.A. Given v and α, u and g are independent to each other. Thus, we split the likelihood into two parts as follows:

(4) $$\begin{array}{ccc} p(u,g|v,\alpha ) & = & p(u|v,\alpha )\cdot p(g|v,\alpha ) \end{array}$$

(5) $$\begin{array}{ccc} p(u|v,\alpha ) & \propto & p(u|v)\\ & = & N(u-v|0,\xi_{1}) \end{array}$$

(6) $$\begin{array}{ccc} p(g|v,\alpha ) & = & N(v-g\alpha |0,\xi_{2}) \end{array}$$

where $N(u-v|0,\xi_{1})$ is a Gaussian distribution with zero mean and variance $\xi_{1}$, $N(v-g\alpha |0,\xi_{2})$ has the same definition as $N(u-v|0,\xi_{1})$, and $\xi_{1}$ and $\xi_{2}$ represent random Gaussian noise and random error in (1) and (2). Based on the noise model in (1), u is independent of α, and thus α is removed in (5), and the p(u|v) is defined as the Gaussian distribution in (5). Based on the discrepancy model in (2), we define p(g|v,α) as the Gaussian distribution in (6).

Second, we define the joint prior distribution of p(v,α). In (2), without considering g, v should be independent to α. The joint prior distribution of p(v,α) is separated into two parts as follows:

(7) $$\begin{array}{ccc} p(v,\alpha ) & = & p(v)\cdot p(\alpha ) \end{array}$$

where p(v) and p(α) are the priors of v and α. Then, to preserve the local contrast from the NIR data and remove noise in the scale map, we define the prior of the wavelet scale map α, which consists of two parts: (1) the prior of its magnitude $p_{lc}\left(\alpha \right)$; (2) the prior of its gradient $p_{gd}\left(\partial^{*}\alpha \right)$ ($\partial^{*}\alpha \in \{\frac{\partial \alpha }{\partial x},\frac{\partial \alpha }{\partial y}$}, $\frac{\partial \alpha }{\partial x}$ and $\frac{\partial \alpha }{\partial y}$ are the partial derivatives of α) as follows:

(8) $$\begin{array}{ccc} p(\alpha ) & = & p_{lc}\left(\alpha \right)\cdot p_{gd}\left(\partial \alpha \right) \end{array}$$

(9) $$\begin{array}{ccc} p_{lc}\left(\alpha \right) & = & N(\alpha |\alpha_{0},\xi_{3}) \end{array}$$

(10) $$\begin{array}{ccc} p_{gd}\left(\partial^{*}\alpha \right) & = & N(\frac{\partial \alpha }{\partial x}|0,\xi_{4x}\left(\frac{\partial g}{\partial x}\right)\cdot \\ & & N(\frac{\partial \alpha }{\partial y}|0,\xi_{4y}\left(\frac{\partial g}{\partial y}\right)) \end{array}$$

where $p_{lc}\left(\alpha \right)$ is defined as the Gaussian distribution with the mean of $\alpha_{0}$ and the variance of $\xi_{3}$. This term is used as the local contrast preservation term that transfers the high contrast and visibility of NIR image to the fusion result. $\alpha_{0}$ is the directive contrast from NIR components, which selects the high contrast component from the NIR wavelet coefficients. Moreover, we define $p_{gd}\left(\partial^{*}\alpha \right)$ as the gradient denoising term that utilizes the gradients of the NIR component as the guidance for adaptive noise removal in wavelet scale map estimation of the high-pass band. $\xi_{4x}$ and $\xi_{4y}$ are defined as the function of the gradient of the NIR components for denoising. $\frac{\partial g}{\partial x}$ and $\frac{\partial g}{\partial y}$ are the partial derivatives of g.

Finally, the prior of the fusion wavelet coefficient can be modeled as the heavy tailed distributions for noise removal, such as the Laplacian distribution and Generalized Gaussian distribution [22]. In this work, the prior p(v) is defined as zero-mean Laplacian with the scaling parameter as follows:

(11) $$\begin{array}{ccc} p(v) & = & \frac{1}{2\xi_{5}}e^{-\frac{\left\|v\right\|}{\xi_{5}}} \end{array}$$

where $\xi_{5}$ is the scaling parameter for the Laplacian distribution.

Based on (1)–(11), we perform MAP estimation by the minimization of energy function as follows:

(12) $$\begin{array}{c} E\left(v,\alpha \right)=\omega_{1}{\left\|v-u\right\|}^{2}+\omega_{2}{\left\|v-g\alpha \right\|}^{2}+\omega_{3}{\left\|\alpha -\alpha_{0}\right\|}^{2}\\ +\omega_{4x}\|\frac{\partial \alpha }{\partial x}{\|}^{2}+\omega_{4y}\|\frac{\partial \alpha }{\partial y}{\|}^{2}+\omega_{5}\left\|v\right\| \end{array}$$

where the parameters $\omega_{1}$–$\omega_{5}$ in (12) are the inverse of the variances $\xi_{1}$–$\xi_{4}$ and the scaling parameter $\xi_{5}$, i.e., $\omega_{1,2,5}=\xi_{1,2,5}^{-1}$, $\omega_{3,4x,4y}=-\xi_{3,4x,4y}^{-1}$.

### 2.3. Parameter Description

The parameters of each term in (12) are described as follows:

#### 2.3.1. Parameters $\omega_{1}$ and $\omega_{2}$

The first term and the second term are the fusion weight of the NIR and RGB wavelet coefficients. We set $\omega_{1}=\omega_{2}=0.5$ for fusion.

#### 2.3.2. Parameters $\omega_{3}$ and $\alpha_{0}$

The third term represents the contrast preservation term in the wavelet scale map estimation. In the high-pass band, we obtain the directive contrast $\alpha_{0}$ using Weber’s law, i.e., the ratio of the Laplacian gradients in the high frequency subband to the local luminance in the low pass subband [23], as follows:

(13) $$\begin{array}{c} \alpha_{0}\left(x,y\right)=\left\{\begin{array}{c} {\left(\frac{1}{g_{l}\left(x,y\right)}\right)}^{\gamma }\cdot \frac{SML(x,y)}{g_{l}\left(x,y\right)}\;if\;g_{l}\left(x,y\right)\neq 0\\ \;SML\left(x,y\right)\;\;if\;g_{l}\left(x,y\right)=0 \end{array}\right. \end{array}$$

where SML(x,y) is the sum-modified-Laplacian gradient [23], $g_{l}\left(x,y\right)$ is the local luminance in the base subband and γ is the visual sensitivity to luminance that ranges from 0.6 to 0.7. We apply the visibility map to the parameter $\omega_{3}$ for transferring the NIR component more than the RGB component as follows:

(14) $$\begin{array}{ccc} \omega_{3} & = & \tau \cdot \phi (VI_{NIR}/VI_{RGB}|\sigma_{1},\gamma_{1}) \end{array}$$

where the visibility map VI is generated by [24], which evaluates the signal visibility of the human visual system (HVS) in the wavelet domain, $\phi (\cdot |\sigma_{1},\gamma_{1})$ uses the wavelet shrinkage function  [1] with parameters $\sigma_{1}$ and $\gamma_{1}$ as the transfer function to compress the dynamic range into [0,1], τ is the constant value, which is set to $10^{-4}$.

Severe noise degrades the main structure of the visibility map in high-pass bands, which significantly affects the performance of the contrast preservation term. We use the relative total variation [25] to reduce the noise of VI and produce the smoothing weight $\omega_{3}$ for structure preservation. The ratio of $VI_{NIR}$ to $VI_{VIS}$ determines the visibility of NIR components over RGB components, i.e., a larger ratio, the more visible the contrast from the NIR components. Thus, a larger ratio, i.e., a larger $\omega_{3}$, provides more contrast transfer from the NIR to RGB components. Here, we set $\sigma_{1}=0.5$ and $\gamma_{1}=2$.

#### 2.3.3. Parameters $\omega_{4x}$ and $\omega_{4y}$

The fourth term considers the use of the gradients of NIR coefficients to guide denoising for the wavelet scale map. $\omega_{4x}$ and $\omega_{4y}$ are defined as follows:

(15) $$\begin{array}{ccc} \omega_{4x} & = & \lambda \cdot (∥\frac{\partial g}{\partial x}\cdot \alpha {{∥}^{\beta }+\varepsilon )}^{-1} \end{array}$$

(16) $$\begin{array}{ccc} \omega_{4y} & = & \lambda \cdot (∥\frac{\partial g}{\partial y}\cdot \alpha {{∥}^{\beta }+\varepsilon )}^{-1} \end{array}$$

where λ, β, and ε are the parameters of wavelet scale map denoising. We set $\lambda =10^{-(M-1)}\cdot \omega_{3}/\tau$ and *M* are the decomposition level. λ is related to the maximum decomposition level *M*. $\omega_{3}$ is used to adaptively control the weight (i.e., $\omega_{4x}$ and $\omega_{4y}$) of gradient denoising. Large $\omega_{3}$ provides coarse filtering (i.e., high $\omega_{4}$ value) of the wavelet scale map, which selects whole regions of visible NIR pixels and then transfers them to RGB ones. Small $\omega_{3}$ (i.e., low $\omega_{4}$) achieves the careful filtering along the gradients of NIR wavelet coefficients, which makes them close to RGB data. Thus, $\omega_{3}$ is large for visible NIR data and small for visible RGB data.

#### 2.3.4. Parameters $\omega_{5}$

The fifth term enforces the denoising of desirable RGB components *v*. We define the adaptive weights $\omega_{5}$ by the adjusted NIR components as:

(17) $$\omega_{5}=\eta \cdot e^{-\frac{g\cdot \alpha }{avg}\left(g\cdot \alpha \right)}$$

where η is the parameter from 0.01 to 0.001 and avg(·) is the average function. $\omega_{5}$ controls the denoising degree based on the adjusted NIR wavelet coefficient. The large magnitude of the adjusted NIR components means more visibility of the NIR data, which is transferred to the fusion result. Thus, it enforces weak denoising (i.e., small $\omega_{5}$ value) for the fusion result because there is less noise in the NIR data. In contrast, a smaller adjusted NIR component means less fusion from the NIR data and, thus, stronger denoising (i.e., a larger $\omega_{5}$ value) due to noisy RGB data.

### 2.4. Numerical Solution

We obtain the latent image by both the fusion and denoising of RGB and NIR images. MFD is iteratively performed by estimating α and v as follows:

**Optimizing α**: with the fixed v, α is calculated with the minimization of energy function E(α) as follows:

(18) $$\begin{array}{c} E\left(\alpha \right)=\omega_{2}{\cdot ∥v-g\alpha ∥}^{2}+\omega_{3}\cdot {∥\alpha -\alpha_{0}∥}^{2}\\ +\omega_{4x}\cdot ∥\frac{\partial \alpha }{\partial x}{∥}^{2}+\omega_{4y}\cdot {∥\frac{\partial \alpha }{\partial y}∥}^{2} \end{array}$$

Based on the parameter design section, we rewrite (18) using the matrix notation as follows:

(19) $$\begin{array}{c} \alpha =\min_{\alpha }\{\omega_{2}{\left(v-g\alpha \right)}^{T}\left(v-g\alpha \right)+{\left(\alpha -\alpha_{0}\right)}^{T}\omega_{3}\left(\alpha -\alpha_{0}\right)\\ +\alpha^{T}D_{x}^{T}\omega_{4x}D_{x}\alpha +\alpha^{T}D_{y}^{T}\omega_{4y}D_{y}\alpha \} \end{array}$$

where v, α, and $\alpha_{0}$ are the vector forms of *v*, α, and $\alpha_{0}$, respectively; g, $\omega_{3}$, $\omega_{4x}$, and $\omega_{4y}$ are the diagonal matrices of the original variables (*g*, $\omega_{3}$, $\omega_{4x}$, and $\omega_{4y}$); Dx and $D_{y}$ are forward difference operators, respectively, and thus $D_{x}^{T}$ and $D_{y}^{T}$ are backward difference operators. We use a solver [26] to minimize (19). We obtain α by minimizing the energy function in (19); thus, the solution of the linear system as follows:

(20) $$\begin{array}{c} (\omega_{2}g^{T}g+\omega_{3}+D_{x}^{T}\omega_{4x}D_{x}+D_{y}^{T}\omega_{4y}D_{y})\alpha \\ =(\omega_{2}g^{T}v+\omega_{3}^{T}\alpha_{0}) \end{array}$$

(21) $$\begin{array}{c} \alpha ={\left(\omega_{2}g^{T}g+\omega_{3}+D_{x}^{T}\omega_{4x}D_{x}+D_{y}^{T}\omega_{4y}D_{y}\right)}^{-1}\\ \left(\omega_{2}g^{T}v+\omega_{3}^{T}\alpha_{0}\right) \end{array}$$

**Optimizing v**: with the fixed α, v is calculated by minimizing E(v) as follows:

(22) $$E\left(v\right)=\omega_{1}{∥v-u∥}^{2}+\omega_{2}{∥v-g\alpha ∥}^{2}+\omega_{5}∥v∥$$

The solution of v in (22) is the soft-thresholding function as follows [1,27]:

(23) $$\begin{array}{ccc} \hat{v} & = & \omega_{1}u+\omega_{2}g\alpha \\ v & = & \mathrm{sign}\left(\hat{v}\right)\max(∥\hat{v}∥-\omega_{5},0) \end{array}$$

where max(·) is the max function and sign· is the sign function, which is defined as x=1 when x>0, x=−1 when x<0 and x=0 when x=0.

### 2.5. Application to Low Pass Fusion

In the low pass subband, we employ the same MFD framework to fuse the NIR and RGB components. However, the denoising term is removed because there is less noise in the low-pass band. (19) and (23) are simplified as follows:

(24) $$\begin{array}{c} \alpha ={\left(\omega_{2}g^{T}g+\omega_{3}\right)}^{-1}\left(\omega_{2}g^{T}v+\omega_{3}^{T}\alpha_{0}\right) \end{array}$$

(25) $$v=\omega_{1}u+\omega_{2}g\alpha$$

(26) $$\alpha_{0}\left(i\right)=R_{i}g\left(i\right)/avg\left(R_{i}g\right)$$

where $\alpha_{0}$ is the directive contrast of RGB components in the low-pass band, which is defined as the ratio of the center pixel intensity to average intensity in the window, avg(·) is average function in an window, $R_{i}$ is a matrix that extracts the patch at the ith pixel location from an image, and the visibility degree VI of low-pass band is calculated similar to that in high-pass band. However, we use a luminance adaptation model [24] to calculate JND threshold without considering inter-band and intra-band masking.

### 2.6. Unified MFD Framework for RGB and NIR Image Fusion

As shown in Figure 1, we first apply the color space conversion, which converts RGB color space to the decorrelated color space (we use YCbCr color space) for the degraded RGB images. Then, we decompose luminance channels of RGB and NIR images by DT-CWT. Next, we use the MDF framework to fuse luminance channel of RGB images and NIR images. The fused luminance image is produced by inverse DT-DWT. Then, we employ a guided filter [28] to denoise the luminance and chrominance channels, and the denoised RGB image is obtained by inverse color space conversion.

Finally, we enhance colors based on the luminance variation after fusion. Algorithm 1 depicts the MDF framework. $\alpha^{n}$, $\alpha_{0}$, u, g and $v^{n}$ mean the variables in high frequency band, where $\alpha_{l}^{n}$, $\alpha_{l0}$, $u_{l}$, $g_{l}$ and $v_{l}^{n}$ mean the variables in the low-pass band. *M* is the maximum decomposition level chosen as a large value because DT-CWT with a large decomposition level (e.g., 3∼5) extracts much noise in high pass subband, while *N* is maximum iteration number. Figure 2d shows that the proposed method successfully handles the discrepancy problem between RGB and NIR images while achieving the fusion result with noise reduction and good contrast based on the NIR image.

**Figure 3:**
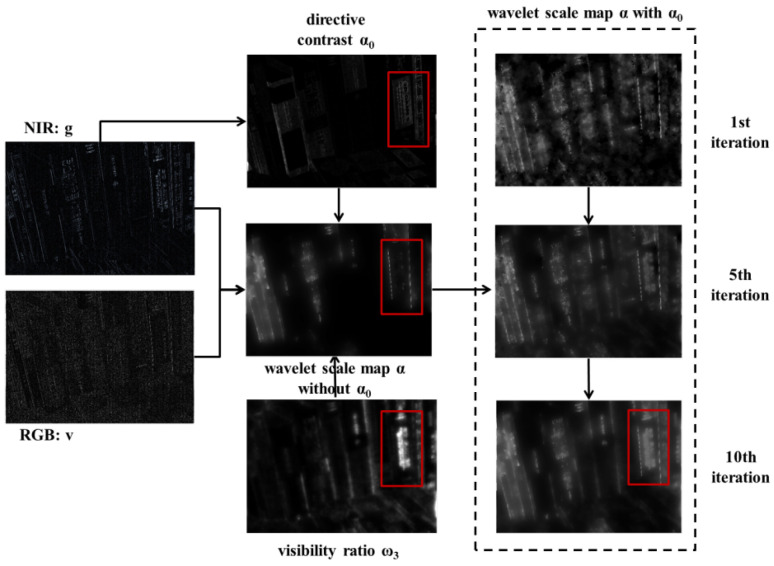
Wavelet scale map generation.

We provide the results of wavelet scale map estimation, and MFD framework for high pass and low-pass bands. Figure 3 shows the generation process for wavelet scale map in one wavelet subband. $\omega_{3}$ represents the visibility of NIR wavelet coefficients compared to RGB components. $\alpha_{0}$ represents the local contrast from NIR components. The wavelet scale map α without $\alpha_{0}$ calculates the correlation (i.e., ratio) of wavelet coefficients between NIR and RGB components to deal with the discrepancy problem. The gradient denoising term guided by NIR wavelet coefficients reduces noise in the scale map and thus main structure in NIR image appears in the scale map. Then, α adjusts wavelet coefficients of NIR image to be compatible with RGB components.

**Figure 4:**
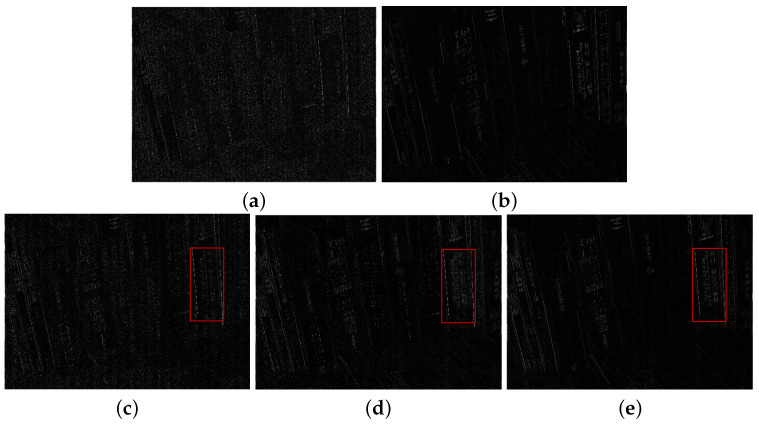
Fusion and denoising results of the high-pass bands in *Books*. (**a**) RGB image. (**b**) NIR image. (**c**) First iteration. (**d**) Third iteration. (**e**) Tenth iteration.

**Figure 5:**
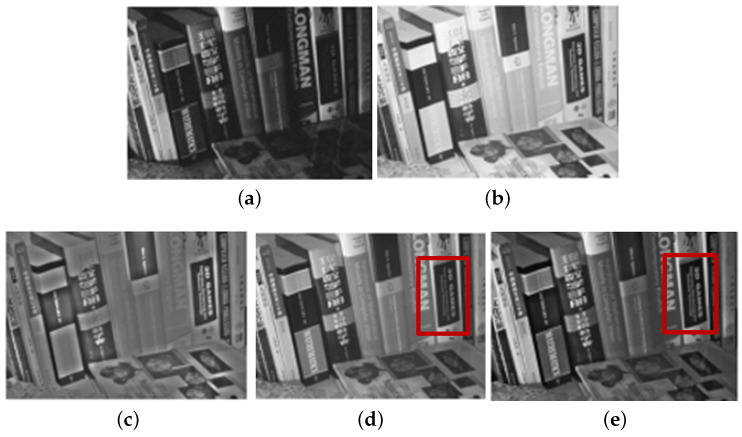
Fusion results of low-pass bands in *Books*. (**a**) RGB image. (**b**) NIR image. (**c**) $\alpha_{0}$. (**d**) Fusion without α. (**e**) Fusion with α.

Guided by the local contrast $\alpha_{0}$ and visibility $\omega_{3}$, we select high contrast and visibility regions from NIR components by α (see the red blocks in Figure 3). Figure 4 shows the fusion and denoising results of NIR and RGB wavelet coefficients in high-pass band. We demonstrated that the fused results by the proposed method contained fine details from the NIR components (see the red blocks in Figure 4). Figure 5 shows the low-pass band fusion results.
**Algorithm 1**  Multi-scale fusion and denoising of NIR and RGB images.**Input:** Noisy gray image from RGB image, NIR image
**Initialize:** $\omega_{1}=\omega_{2}=0.5$,$\sigma_{1}=0.5$,$\gamma_{1}=2$, η=0.005 β=1.2, ε=0.001, $\alpha^{0}=I$, $v^{0}=u$ M=3∼5, N=20, $\tau =10^{-4}$, $\varepsilon_{1}=\varepsilon_{2}=10^{-2}$. **1.** Perform DT-CWT on noisy gray and NIR images. **2. Detail layer:** **For** *m*=1:*M* (*M*: Maximum decomposition)    **For** *n*=1:*N* (*N*: Maximum iteration number)        *a.* Calculate VI of $v^{n}$ and g [24];        *b.* Calculate $\alpha_{0}$ from g by (13);        *c.* Calculate $\omega_{3}-\omega_{5}$ by (14)–(17);        *d.* Optimize $\alpha^{n+1}$ by (21);        *e.* Optimize $v^{n+1}$ by (23);        **if** $∥\alpha^{n+1}-\alpha^{n}{∥}_{2}^{2}/{∥\alpha^{n}∥}_{2}^{2}<\varepsilon_{1}$ and            $∥v^{n+1}-v^{n}{∥}_{2}^{2}/{∥v^{n}∥}_{2}^{2}<\varepsilon_{2}$; **break**;     **end For** **end For****3. Base layer:****For** *n*=1:*N* (*N*: Maximum iteration number)    *a.* Calculate VI of $v_{l}^{n}$ and $g_{l}$ [24];    *b.* Calculate $\alpha_{l0}$ from *g* by (26);    *c.* Calculate $\omega_{3}$ by (14);    *d.* Optimize $\alpha_{l}^{n+1}$ and $v_{l}^{n+1}$ by (24)–(25);    **if** $∥\alpha_{l}^{n+1}-\alpha_{l}^{n}{∥}_{2}^{2}/{∥\alpha_{l}^{n}∥}_{2}^{2}<\varepsilon_{1}$ and        $∥v_{l}^{n+1}-v_{l}^{n}{∥}_{2}^{2}/{∥v_{l}^{n}∥}_{2}^{2}<\varepsilon_{2}$; **break**;**end For****4.** Perform inverse DT-CWT.
**Output:** Fused gray image.


Compared to the fusion without α, the fusion results with α have a similar appearance to the original gray images, which indicates that the fusion results with α suffered less discrepancy artifacts (see Figure 5d,e). Figure 5c shows the local contrast map of the NIR data where bright pixels mean more details were transferred to the RGB components. Thus, the proposed base band fusion with α and $\alpha_{0}$ solved the discrepancy problem and obtained visible details from NIR data as shown in the red blocks of Figure 5d,e.

### 2.7. Chroma Denoising and Color Enhancement

In Section IV.B, we obtained the fused luminance image by combining RGB and NIR images. Then, we employed the guided image filter [28] to denoise the chrominance channels guided by the fused luminance results [29]. The primary idea of [29] is the high correlation in the texture information over color channels. Based on the correlation, [29] transfers the texture information of the fused luma channel to the other two channels and removes the noise around the texture by the guided filter. Meanwhile, the guided filter is employed to remove noise in the noisy luma channel. Then, the denoised luma and chroma channels are combined and inverse color space conversion is used to obtain the denoised original images. Finally, we enhance the color of the fused image based on the variation of luminance after fusion as follows [30]:

(27) $$M_{e}\left(x,y\right)={\left(\frac{Y^{\prime }\left(x,y\right)}{Y^{d}\left(x,y\right)}\right)}^{\beta }\cdot M_{d}\left(x,y\right)$$

where $M_{e}\left(x,y\right)$ and $M_{d}\left(x,y\right)$ are trichromatic (three channels, i.e., R,G,B channels) channel values of the output color and denoised images, respectively; $Y^{\prime }\left(x,y\right)$ and $Y^{d}\left(x,y\right)$ are gray images from the fused result and the denoised original image, respectively; and β is the sensitive factor whose range is [0.6, 1.0]. In (27), we utilize the ratio of luminance variation to enhance the three channels independently.

**Figure 6:**
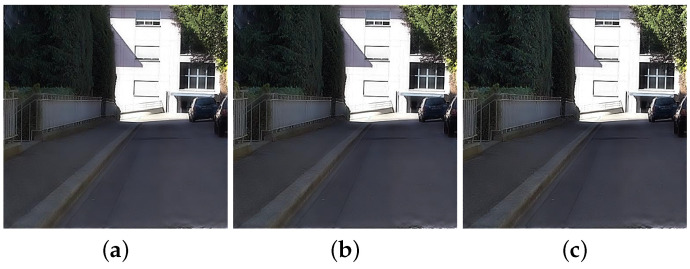
Color enhancement results with (**a**) β=0.6, (**b**) β=0.8, and (**c**) β=1.0.

**Figure 7:**
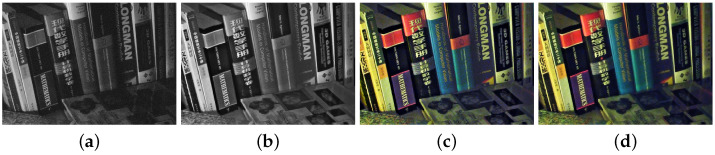
Fusion and color enhancement results in *Books*. (**a**) Original gray image. (**b**) Fusion results in the luma channel. (**c**) Inverse color space conversion (YCbCr → RGB). (**d**) Color enhancement results.

As a result, the colors of the fusion images are enhanced more with the increase of β (see the trees in Figure 6). Figure 7 shows the fusion and color enhancement results of the proposed method. The proposed method produces fusion results with noise removal and detail transferring from the NIR images (see Figure 7a,b). Color enhancement provides saturated colors in images compared to the direct inverse color space conversion (see Figure 7c,d).

## 3. Experimental Results

### 3.1. Multi-Spectral Fusion of NIR and RGB Images

**Figure 8:**
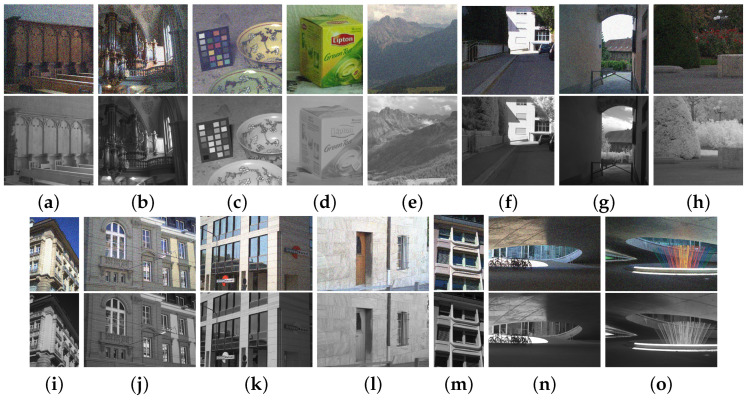
Test image pairs with noisy RGB images (**top**) and NIR images (**bottom**).

In the experiments, we used fifteen pairs of RGB and NIR images obtained from [7,10,15] as shown in Figure 8. Figure 8a–d are from [15], Figure 8e–h are from [7], and Figure 8i–o are from [10]. We added Gaussian noise to the RGB images in Figure 8e–o. The RGB and NIR image pairs are well registered with the same spatial resolution from 436×512 to 1147×800. For the tests, we used a PC with Intel (R) Core (TM) 547 i5 CPU (2.60 GHZ) and 4.00 GB RAM running a Windows environment and MATLAB 2012b. For deep learning methods, we used Nvidia GTX2080Ti with the Ubuntu 16.04 environment.

**Figure 9:**
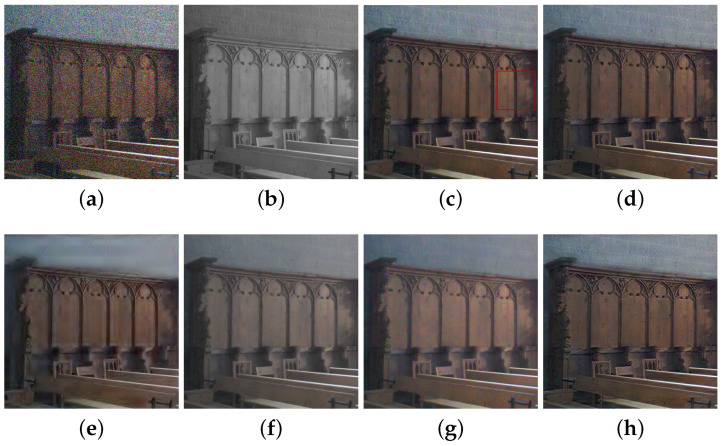
Experimental results for a test image. (**a**) Noisy RGB image. (**b**) NIR image. (**c**) DWLS [11]. (**d**) SM [8]. (**e**) GWS [1]. (**f**) DenseFuse [17]. (**g**) UDIF [20]. (**h**) Proposed method.

**Figure 10:**
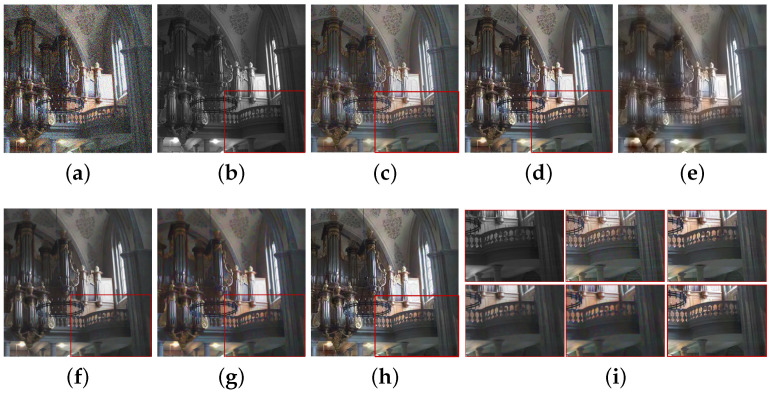
Experimental results for a test image. (**a**) Noisy RGB image. (**b**) NIR image. (**c**) DWLS [11]. (**d**) SM [8]. (**e**) GWS [1]. (**f**) DenseFuse [17]. (**g**) UDIF [20]. (**h**) Proposed method. (**i**) Zoomed regions of red blocks (From top left to bottom right: (**b**–**d**,**f**–**h**)).

**Figure 11:**
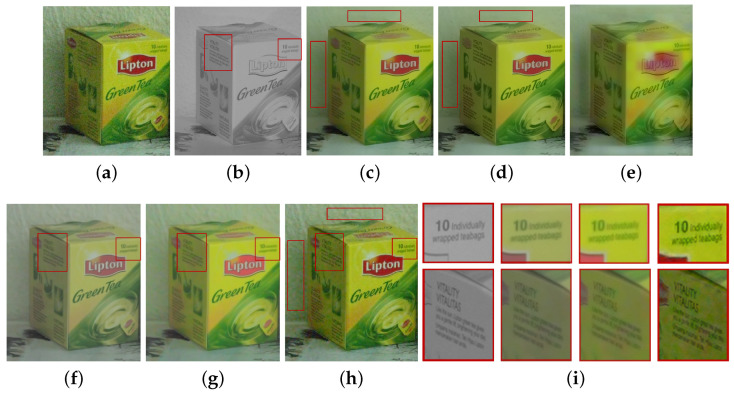
Experimental results for a test image. (**a**) Noisy RGB image. (**b**) NIR image. (**c**) DWLS [11]. (**d**) SM [8]. (**e**) GWS [1]. (**f**) DenseFuse [17]. (**g**) UDIF [20]. (**h**) Proposed method. (**i**) Zoomed regions of red blocks (From Left to Right: (**b**,**f**–**h**).

**Figure 12:**
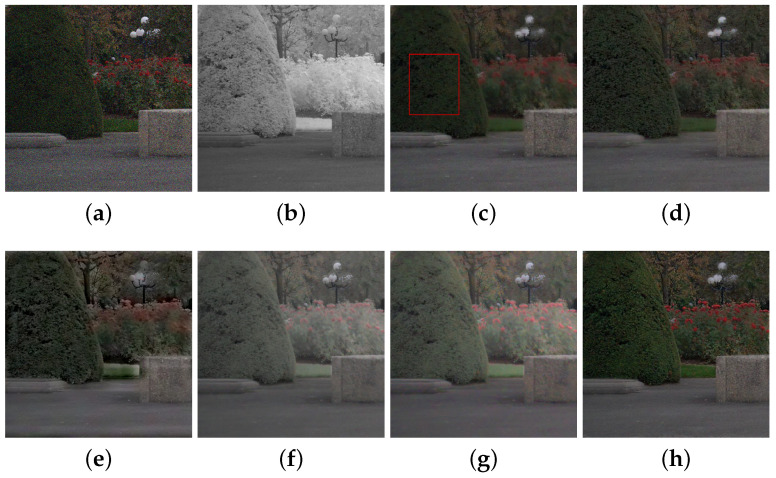
Experimental results for a test image. (**a**) Noisy RGB image. (**b**) NIR image. (**c**) DWLS [11]. (**d**) SM [8]. (**e**) GWS [1]. (**f**) DenseFuse [17]. (**g**) UDIF [20]. (**h**) Proposed method.

We compared the performance of the proposed method with those of the guided wavelet shrinkage (GWS) [1], dual WLS smoothing (DWLS) [11], scale map (SM) [8], DenseFuse [17], and Unsupervised Deep Image Fusion using structure tensor (UDIF) [20]. To conduct the experiments, we first removed noise using BM3D [31], and then we employed DenseFuse and UDIF for fusion. Figure 9, Figure 10, Figure 11 and Figure 12 show the experimental results from different methods.

GWS [1] directly fuses RGB and NIR images without considering discrepancies, and thus there is much contrast attenuation in the results as shown in Figure 9e, Figure 10e, Figure 11e, and Figure 12e. DWLS [11] uses the gradients of the NIR and Y channels of the initially denoised RGB images as the guidance for WLS smoothing and, then, transfers the details of NIR data to the smoothing results. However, the results seem blurred in the details because the initial denoising smoothes some details of the RGB data (see the red blocks in Figure 11c and Figure 12c), and contains some noise (see the red blocks in Figure 9c and Figure 10c).

SM [8] uses the scale map to correct the discrepancy of common edges between RGB and NIR images; however, some different details between two images seem blurred and are lost in the results (see the red blocks in Figure 10d and Figure 11d). The zoomed red blocks in Figure 10d are provided in Figure 10i. DenseFuse [17] introduces the dense blocks in an encoder–decoder framework and preserves more extracted features of the middle layer. However, the generated results causes serious color distortion (see Figure 9, Figure 10, Figure 11 and Figure 12f) and loss of details (see the red blocks of Figure 10 and Figure 11f and the zoomed regions in Figure 10 and Figure 11i).

UDIF [20] is an unsupervised deep learning fusion network with a structure tensor loss. They lead to the attenuated details (see the red blocks in Figure 10 and Figure 11g and the zoomed regions in Figure 10 and Figure 11i). Color distortion happens in the results (see Figure 12g). The proposed method adjusts the NIR coefficients to have the same distribution as RGB data and obtain a wavelet scale map for fusion. Thus, our method effectively handles the discrepancy between RGB and NIR images and produces both fusion and denoising without contrast attenuation compared to GWS (see Figure 9, Figure 10, Figure 11 and Figure 12e,h).

Moreover, the contrast preservation term and visibility ratio detect high and visible contrasts from the NIR wavelet coefficients and then transfer them to RGB components. Thus, our results successfully preserve visible local contrast from the NIR data (see Figure 9, Figure 10, Figure 11 and Figure 12h). The proposed method reduces more noise and artifacts compared with DWLS (see Figure 9Figure 10c,h, and the zoomed region in Figure 10i) because of the Laplacian distribution modeling of the fusion wavelet coefficients. As shown in Figure 11, the proposed method preserves the local contrast of RGB images better than DWLS and SM (see the red blocks on the wall of Figure 11c,d,h).

This is because the low visibility ratio on the wall, i.e., the low visibility degree in the NIR data, reduces the effect of the contrast preservation term. As the gradient denoising term provides gradient enhancement for the scale map guided by the NIR wavelet coefficients, the proposed method is very effective in enhancing the details in the boxes compared to DenseFuse and UDIF (see the red blocks on the boxes in Figure 11f–h and its zoomed regions in Figure 11i).

As shown in Figure 12, the proposed method achieved better performance in detail preservation and color reproduction compared with the other methods. Finally, the proposed method generates the fusion results with vivid colors compared to DenseFuse and UDIF (see Figure 9, Figure 11 and Figure 12f–h) because we enhance the colors corresponding to the luminance variation.

Moreover, we performed the quantitative measurements in terms of the discrete entropy (DE) [32], feature-based blind image quality evaluator (FBIQE) [33], and color image quality (CIQ) [34]. DE [32] is defined as

(28) $$H\left(p\right)=-\sum_{L-1}^{i=0}p\left(i\right)log_{2}\left(p\left(i\right)\right)$$

where p(i) is the probability density function at the intensity level *i* and *L* is maximum pixel value (L=255). DE estimates the detail amount of an image based on the histogram distribution. FBIQE measures the modified Bhattacharyya distance between the natural statistics of distorted images and the reference naturalness statistics in terms of the local structures, contrast, multiscale decomposition, and colors [33]. FBIQE is calculated as

(29) $$q=\sqrt{{\left(\mu -\mu^{\prime }\right)}^{T}\left(\frac{\sigma +\sigma \prime }{2}\left(\mu -\mu \prime \right)\right)}$$

where (μ,Σ) and $\left(\mu^{\prime },\Sigma^{\prime }\right)$ represent the mean vector and variance matrix of natural statistics features from the test and reference images. The natural statistics features are modeled using multivariate Gaussian (MVG) model with (μ,Σ) as follows:

(30) $$f\left(x\right)=\frac{1}{{\left(2\pi \right)}^{m/2}{∥\sigma ∥}^{-1}}\exp\left(-\frac{1}{2}{\left(x-\mu \right)}^{T}\sigma^{-1}\left(x-\mu \right)\right)$$

where *m* is the dimension of the vector μ. The color image quality (CIQ) [34] metric assesses the image quality in terms of sharpness, colorfulness, and contrast characteristics. In the CIQ metric, the sharpness, colorfulness, and contrast measurement are calculated first and then combined with multiple linear regression (MLR) as follows:

(31) $$\mathrm{CIQ}=c_{1}\times \mathrm{colorfulness}+c_{2}\times \mathrm{sharpeness}+c_{3}\times \mathrm{contrast}$$

where $c_{1}$, $c_{2}$, and $c_{3}$ are constants [34].

A larger DE indicates more details in the fused image, while a smaller FQBIE indicates good image quality with less noise, a clearer structure, and more natural colors in the fused images. Larger CIQ values indicate better image quality in the results with high contrast, sharp structure, and saturated colors. Table 1 shows the quantitative measurements on five methods.

The proposed method achieved the best performance in the average DE because the gradient denoising term performs gradient enhancement in a wavelet scale map guided by the gradients of the NIR wavelet coefficients. The detail-enhanced wavelet scale map provides detail enhancement of the fused RGB images. In addition, the proposed method achieved the best performance in FBQIE because the proposed method produced a fused RGB image with less noise and better details. Due to the good color enhancement and gradient enhancement in the proposed method, the fusion results achieved the best performance in the CIQ metric.

### 3.2. Parameter Analysis

**Figure 13:**
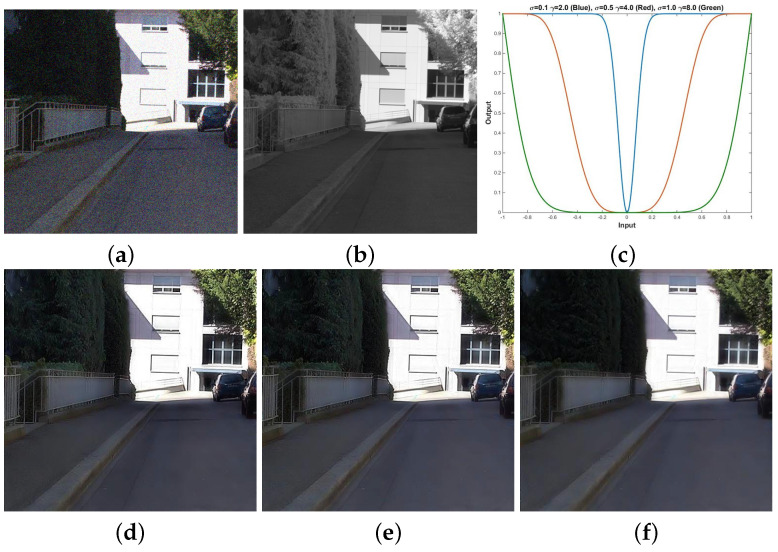
Fusion results from the MFD framework with different $\sigma_{1}$ and $\gamma_{1}$ pairs in the contrast preservation term. (**a**) Noisy RGB image. (**b**) NIR image. (**c**) Transfer function (blue, red, and green curves corresponding to (**d**–**f**)). (**d**) $\sigma_{1}=0.1$ and $\gamma_{1}=2.0$. (**e**) $\sigma_{1}=0.5$ and $\gamma_{1}=4.0$. (**f**) $\sigma_{1}=1.0$ and $\gamma_{1}=8.0$.

**Figure 14:**
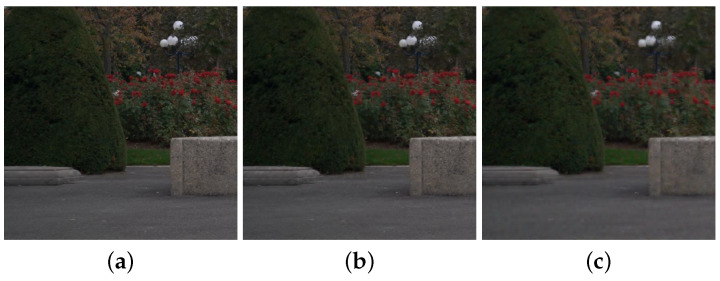
Fusion results from the MFD framework with different λ and β in the gradient denoising term for wavelet scale map estimation. (**a**) β=1.2 and λ with visibility ratio; (**b**) β=2.0 and λ with visibility ratio. (**c**) β=2.0 and λ without visibility ratio. The visibility ratio means $VI_{NIR}/VI_{RGB}$ in (14).

We provide the effects of key parameters on the fusion results by the proposed method as follows. First, we perform the experiments to evaluate the effects of $\sigma_{1}$ and $\gamma_{1}$ (in the contrast preservation term) on the fusion performance as shown in Figure 13. We observed that the fusion results contained few NIR data with the increase of $\sigma_{1}$ and $\gamma_{1}$. This is because increasing $\sigma_{1}$ and $\gamma_{1}$ converts most of the visibility ratio value (see (14)) into 0 as shown in Figure 13c. Thus, few NIR data are transferred to the fusion result. We assess the effects of λ and β (in the gradient denoising term) on the fusion result as shown in Figure 14.

**Figure 15:**
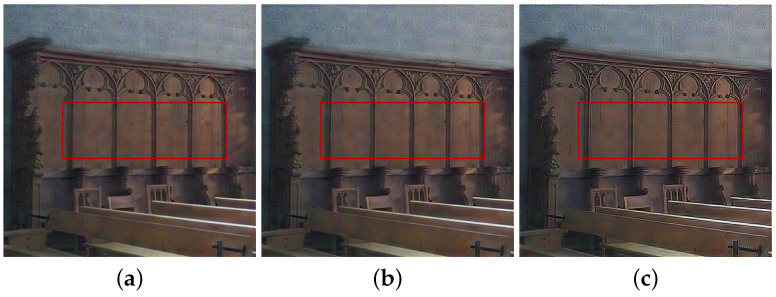
Fusion results from the MFD framework with different η in the denoising term for fused RGB image. (**a**) η=0.001. (**b**) η=0.005. (**c**) η=0.01.

From the figures, increasing β leads to the blur effects (see Figure 14a,b) because a large β reduces the gradient magnitude. By introducing the visibility ratio into λ, the fusion result contains sharp details (see Figure 14b,c) because the visibility ratio adaptively selects the NIR texture transfer and gradient denoising. A low visibility ratio enforces the gradient enhancement in gradient denoising terms. Finally, we provide experiments on the effect of η in the denoising term for the fusion result as shown in Figure 15. We concluded that the results with large η distorted the image details (see the red blocks) because a large η set the small wavelet coefficients of the fusion result to 0 in (23).

### 3.3. Comparison between Different Wavelet Transforms

**Figure 16:**
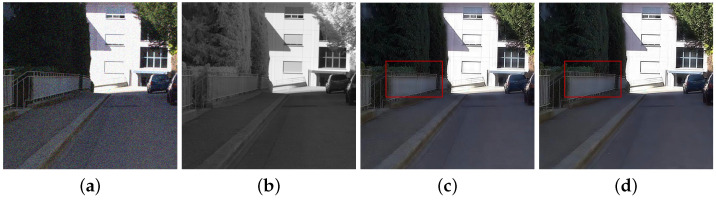
Fusion results from the MFD framework under DWT and DTCWT. (**a**) Noisy RGB image. (**b**) NIR image. (**c**) Fusion under DWT. (**d**) Fusion under DT-CWT.

We implement the proposed method on DWT and DT-CWT decompositions based on the visibility measurements provided by [24,35]. Figure 16 shows the fusion results from the proposed method under DWT and DTCWT. From the figures, the results from DT-CWT contain less artifacts and sharper details compared with DWT (see the red boxes in Figure 16). Thus, we implement the proposed method under DT-CWT.

### 3.4. Comparison with Different Color Spaces

**Figure 17:**
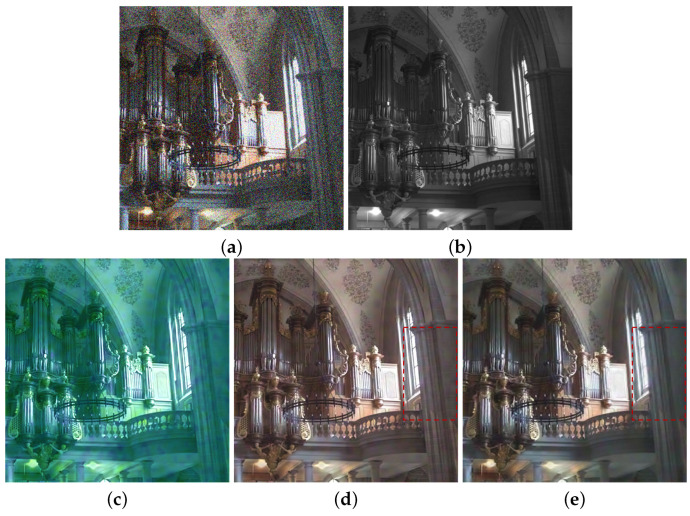
Fusion results from the proposed method on different color spaces. (**a**) Noisy RGB image; (**b**) NIR image; (**c**) Fusion result on HSV; (**d**) Fusion result on CIE LAB; (**e**) Fusion result on YCbCr.

We tested the proposed method on the YCbCr, HSV, and CIELAB color spaces. We used fifteen test images for the experiments in Figure 8. Figure 17 shows the visual comparison among three color spaces by the proposed method. The proposed method in YCbCr color space generated the results with the least color distortion (see the red blocks in Figure 17d,e and the greenish color artifacts in Figure 17c). Table 2 shows the performance comparison among the HSV, CIE LAB, and YCbCr color spaces with the proposed method. The proposed method achieved the best performance in the quantitative measurements. Thus, we adopt the YCbCr color space for the proposed method.

### 3.5. Computational Complexity

In this section, we provide the runtime of the proposed method and the compared methods on Table 3 (image size: 512×512). The average processing time of the proposed method was 14.94 s/image, which was the longest times among the methods. In the future, we will consider improving the speed of the proposed method by optimization in (18).

### 3.6. Application to RGB-NIR Images under Normal Illumination

**Figure 18:**
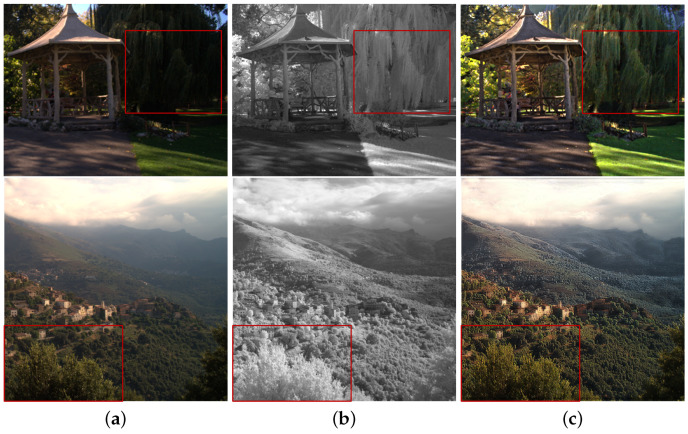
Experimental results for the paired images under normal illumination conditions. (**a**) RGB images; (**b**) NIR images; (**c**) Fusion results.

We applied the proposed method to the paired images captured under a normal illumination condition. As there is little noise in RGB images, we remove the chroma denoising in the proposed method and the denoising term in (23). (23) is rewritten to (25). However, the gradient denoising term is kept because it provides detail enhancement from the visible NIR components in the fusion results. As shown in Figure 18, the proposed method generates the enhanced results with a clearer structure and finer details (see the red blocks in Figure 18).

### 3.7. Fusion of RGB Luminance Channel and NIR Image in a Local Manner

**Figure 19:**
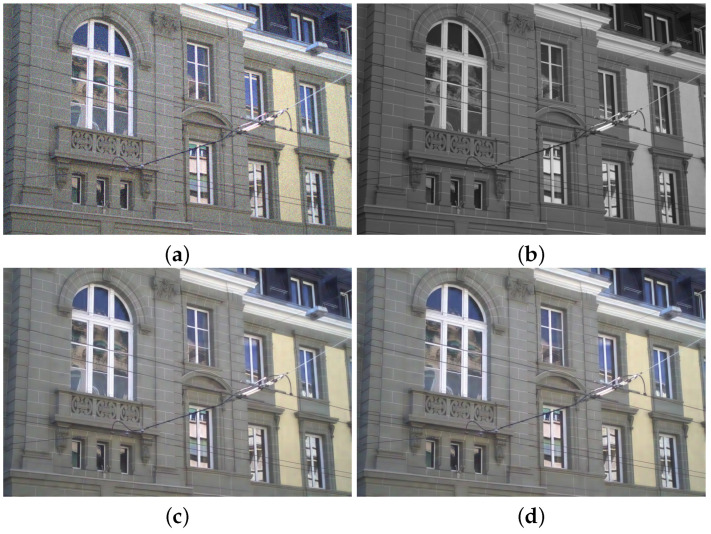
Experimental results from the proposed method in a local manner (the maximum decomposition level is 4). (**a**) RGB image; (**b**) NIR image; (**c**) Fusion result using a 64×64 local window; (**d**) Fusion result using a 128×128 local window.

We provide local scale map estimation and fusion on the RGB luminance channel and NIR image. Chroma denoising and color enhancement are then used to obtain fused color images. In the local scale map estimation and fusion stage, we first divide the RGB luminance channel and NIR image into overlapping blocks. Then, we apply the proposed method to each block pair and estimate the local scale map. We obtain each fusion block $y_{p}^{\prime }$ corresponding to the local scale map. Finally, we combine all overlapping blocks with gaussian kernels as follows:

(32) $$Y_{p}^{\prime }=\frac{\sum_{x}\omega_{p}\left(x\right)\cdot y_{p}^{\prime }\left(x\right)}{\sum_{x}^{\omega_{p}\left(x\right)}}$$

where $Y_{p}^{\prime }$ is the global luminance at the pixel location *p* and $y_{p}^{\prime }\left(x\right)$ is the local fused luminance in a m×m window at the center pixel *p*; *m* is the window size; $\omega_{p}\left(x\right)$ is the weight function, which is inversely proportional to the $l_{2}$ norm distance between the center pixel *p* and the neighbor pixel *x*, which is formulated as follows:

(33) $$\omega_{p}\left(x\right)=\exp\left(-\frac{{\left(x-p\right)}^{2}}{\gamma }\right)$$

where γ is the Gaussian parameter, which is set to 3. The relationship between the minimum size of window *m* and the maximum decomposition level *M* of wavelet is $m=2^{\left(M+1\right)}$. This is because the image patches should be decomposed fully (i.e., the size of last decomposed image should be larger than or equal to 2×2). In this work, we set the maximum decomposition level to 3–5. Thus, the minimum window size corresponds to 16–64. Figure 19 shows the experimental results from the proposed method in a local manner. The proposed method worked on the test images in a local manner and achieved good performance.

### 3.8. Application to RGB-NIR Images with JPEG Compression

**Figure 20:**
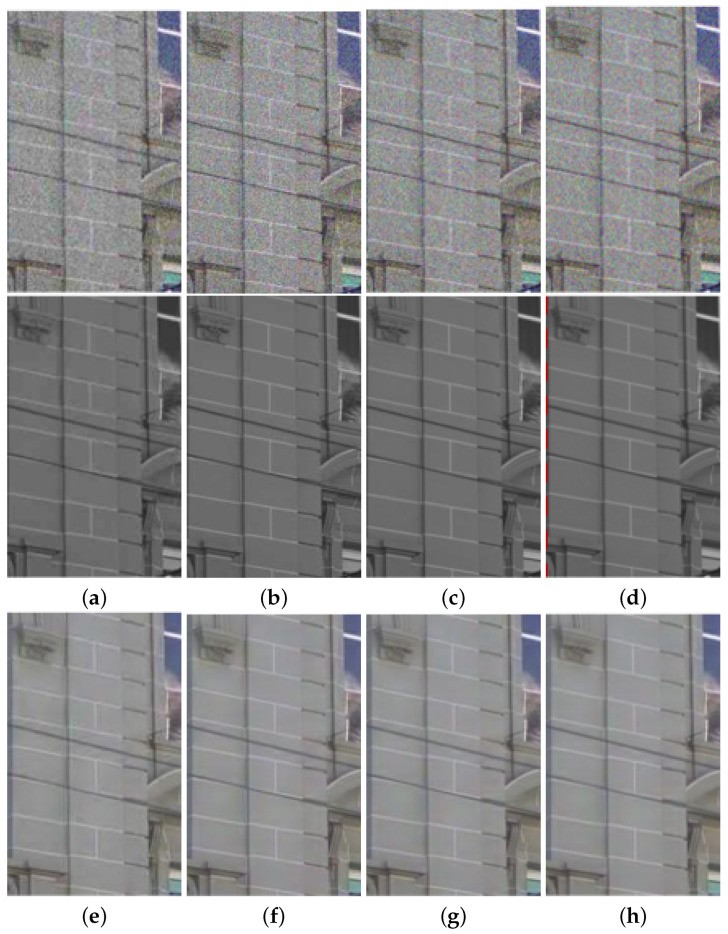
Fusion results from the proposed method on the compressed test images with different compression degrees: (**a**) 0.25; (**b**) 0.5; (**c**) 0.75; (**d**) png format; (**e**) Fusion results for (**a**); (**f**) Fusion results for (**b**); (**g**) Fusion results for (**c**); (**h**) Fusion results for (**d**). (From (**a**) to (**d**), **top**: RGB images; **bottom**: NIR images).

We applied the proposed method to the test images with JPEG compression. We performed JPEG compression on the test images using the *imwrite* function in MATLAB. In the *imwrite* function, we set the compression degree to 0.25, 0.5, and 0.75. In the previous manuscript, the lossless test images had the png format. Figure 20 shows the visual quality of the proposed method on the compressed test images. As shown in Figure 20, in a high compression degree (i.e., 0.25), the ringing artifacts appeared in the NIR images, and noisy blocking artifacts appeared in the RGB images (see Figure 20a,b).

The fusion results contained ringing artifacts (see Figure 20e,f). However, in a low compression degree (i.e., 0.75), the proposed method achieved the same enhancement results on the compressed images as the lossless png format images (see Figure 20g,h). Table 4 shows the performance of the proposed methods on the compressed images with different compression degrees.

From the FBIQE and CIQ metrics, the results were corrupted under the high JPEG compression degree and had good quality under the low JPEG compression degree. This is because ringing artifacts decrease the performance of naturalness and contrast measurements in FBIQE and CIQ metrics. The DE values did not change significantly and were even better under the high compression degree because ringing artifacts appear in the fusion results (see Figure 20e,f), and the DE metric considers the artifacts as the amount of details.

### 3.9. Limitation and Future Work

There are two limitations of the proposed MFD framework. First, we did not consider the calibration problem for RGB and NIR image fusion. The proposed method generates fusion results with artificial edges when the data are not calibrated well. One solution is to combine the affine transform into the proposed MFD method to solve the registration problem [36]. Second, the proposed method does not work well on NIR regions with weak structure.

**Figure 21:**
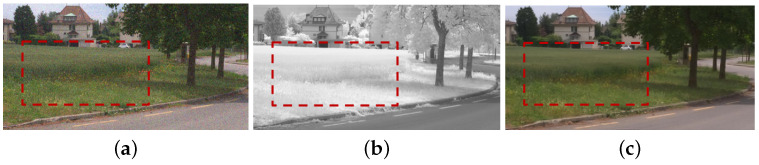
Experimental results on test paired images with weak structure in the NIR images. (**a**) RGB image; (**b**) NIR image; (**c**) Fusion result.

When NIR images contain weak structure and RGB images are seriously corrupted by noise, the weak gradients of the NIR regions smooth the fusion results, and the proposed method attenuates the details in the fusion regions (see the red blocks of grass in Figure 21). The adaptive selection of static denoising (NIR guidance denoising) and dynamic denoising (RGB self-denoising) is one solution to overcome this problem [37].

## 4. Conclusions

In this paper, we proposed MFD of RGB and NIR images based on multi-scale wavelet analysis. We conducted MFD by MAP estimation in the wavelet domain. In the luminance channel, we provided a discrepancy model to deal with the discrepancy between the RGB and NIR images. The discrepancy was obtained by the correlation between the two types of data. We used the priors of the wavelet scale map and its gradient as the contrast preservation term and gradient denoising term, respectively. Then, we adjusted the NIR image based on the scale map to fuse it with the RGB image.

The prior of the fusion wavelet coefficients was modeled as the Laplacian distribution with an adaptive scaling parameter based on the adjusted NIR image to reduce noise. In the chrominance channels, we used the guided filter to denoise the noise with the guidance of the fused luminance. Finally, we employed color enhancement based on the variation of the luminance after the fusion process. Our experimental results demonstrated that the proposed method achieved excellent fusion performance with clear structure and good details.

## Figures and Tables

**Table 1:** Performance comparison between GWS [1], DWLS [11], SM [8], DenseFuse [17], UDIF [20], and the proposed method.

Metrics	DWLS	SM	GWS	Dense.	UDIF	Pro.
DE	7.082	7.046	7.030	6.841	6.865	**7.128**
FBIQE	29.639	27.269	27.909	29.600	30.469	**26.836**
CIQ	0.912	0.914	0.904	0.788	0.841	**0.961**

Bold numbers represent the best performance in each metric.

**Table 2:** Performance comparison among the HSV, CIE LAB, and YCbCr color spaces with the proposed method.

Metrics	HSV	CIE LAB	YCbCr
DE	7.015	7.032	**7.128**
FBIQE	28.057	29.747	**26.836**
CIQ	0.764	0.896	**0.961**

Bold numbers represent the best performance in each metric.

**Table 3:** Computation time of the six compared methods (image size: 512×512).

Methods	DWLS	SM	GWS	Dense.	UDIF	Pro.
Time(s/image)	3.42	7.99	0.60	0.40	0.29	14.94

**Table 4:** Performance comparison of the proposed method on test images in terms of the DE, FBIQE, and CIQ metrics.

Metrics	0.25	0.5	0.75	png
DE	**7.137**	7.134	7.132	7.128
FBIQE	29.283	27.486	26.895	**26.836**
CIQ	0.803	0.826	0.934	**0.961**

Bold numbers represent the best performance in each metric.

## Data Availability

Publicly available datasets were analyzed in this study. These data can be found here: https://sites.google.com/site/changhwan76son/home/project-icip2015/, https://sites.google.com/site/changhwan76son/home/multi-modal-image-pair-fusion/ as well as https://www.epfl.ch/labs/ivrl/research/downloads/colouring-the-near-infrared/ (accessed on 22 May 2021).
